# Does the use of antenatal corticosteroids reduce respiratory morbidity in babies born in late preterm period?

**DOI:** 10.1186/s12884-024-06304-6

**Published:** 2024-05-02

**Authors:** Khadijah A. Shittu, Bolaji Ahmed, Kabiru Afolarin Rabiu, Fatimat Akinlusi, Oluwarotimi I. Akinola

**Affiliations:** 1 Department of Obstetrics & Gynaecology, Lagos State University Teaching Hospital, Lagos, Nigeria; 2 Programme Department, Damien Foundation Belgium (Nigeria Project), Lagos, Nigeria

**Keywords:** Corticosteroids, Late preterm period, Respiratory morbidity, Antenatal

## Abstract

**Background:**

The aim of this study is to determine the effectiveness of antenatal corticosteroid in reducing respiratory morbidity in babies born in the late preterm period.

**Methods:**

Two hundred and eighty-six pregnant women at risk of having a late preterm delivery were studied. One hundred and forty-three (143) served as the cases and were given 2 doses of 12 mg intramuscular dexamethasone 12 h apart, while 143 served as the controls and were given a similar quantity of placebo. The women were followed up prospectively and data were collected on the pregnant women and their newborns on a standardized form. The neonates were assessed for acute respiratory distress syndrome and transient tachypnea of the newborn based on clinical signs, symptoms, and chest x-ray results (when indicated). The primary outcome was the occurrence of neonatal respiratory morbidity.

**Results:**

The primary outcome occurred in 5 out of 130 infants (3.8%) in the dexamethasone group and 31 out of 122 (25.4%) in the placebo group (*P* value = 0.000003). Birth asphyxia, neonatal intensive care admission and need for active resuscitation at birth also occurred significantly less frequently in the dexamethasone group (*P* value 0.004, 0.009, 0.014 respectively). There were no significant group differences in the incidence of neonatal sepsis, neonatal jaundice, hypoglycemia and feeding difficulties.

**Conclusions:**

Administration of dexamethasone to women at risk for late preterm delivery significantly reduced the rate of neonatal respiratory complications, neonatal intensive care unit admission, and need for active resuscitation at birth.

**Trial registration:**

PACTR (www.pactr.org) Registration Number: PACTR202304579281358. The study was retrospectively registered on April 19, 2023.

## Introduction

Late preterm delivery is the delivery of a neonate between 34 + ^0^ weeks to 36 + ^6^ weeks after the beginning of a woman’s last menstrual period [1]. It accounts for 73% of preterm deliveries [2]. They have similar birth weight with term babies, and are usually assumed to be developmentally mature by parents and caregivers, hence are often managed in nurseries or remain with their mothers after birth, [3] but these late preterm infants are physiologically and metabolically immature and have limited compensatory responses to the extra uterine environment compared with term infants [3, 4]. As a consequence, they are at higher risk than term infants of developing medical complications that result in higher rates of morbidity and mortality [4]. In addition, they have higher risk of hospital readmission during the neonatal period than the term infants [5].

There is evidence for the common use of antenatal corticosteroids in Obstetrics, especially for pregnancies at risk for early preterm deliveries [6]. There is also strong evidence to suggest that antenatal corticosteroids are effective in reducing respiratory distress syndrome when administered to women that are likely to deliver before 34 weeks of gestation [7].

Presently, the use of antenatal corticosteroids is not indicated in women who have high chances of having preterm delivery after 34 weeks [6]. This is because the chances of survival of neonates at 34 to 35 weeks of gestation tend to be very high [8, 9]. However, recent studies have shown that infants who are born during the late preterm period (34 weeks 0 day to 36 weeks 6 days) have more neonatal and childhood complications than newborns who are born at term (37 weeks or later) [3, 4, 10, 11].

Presently, there is no generally accepted consensus on the use of antenatal corticosteroid for the prevention of respiratory morbidity in babies born in the late preterm period. Studies done in Thailand, [12] Sweden, [13] Spain, [14] Iran, [15] and the Antenatal late preterm study, [16] all support the use of antenatal corticosteroids in the prevention of morbidity and mortality associated with late preterm delivery, while those done in Brazil, [17] the USA, [18] India, [19] and the recently published WHO randomized control trial [20] did not show any benefit. One study even showed an increase in perinatal morbidity and mortality in those that had antenatal corticosteroid [20]. Presently, in Nigeria, where the burden of prematurity is high, no study has been done to evaluate the role of antenatal corticosteroid in late preterm delivery, there is therefore need for more studies on this subject.

Most studies have used betamethasone or dexamethasone as the antenatal steroid. Current research has not shown any significant differences in complication or efficacy of both drugs, hence the choice of dexamethasone in this study [21, 22]. Also, the study was done in a low resource setting where dexamethasone is significantly cheaper compared to betamethasone.

This present study is essentially aimed at determining whether the use of antenatal corticosteroids reduces the respiratory morbidity in late preterm babies in the Nigerian population.

## Materials and methods

The study is a double blind, randomized controlled trial of women at risk of late preterm delivery. The Maternal and Child Centre (MCC), an annex of the Obstetrics Department of the Lagos State University Teaching Hospital, located at Ifako Ijaye local government area of Lagos state was the location for this study [23]. The facility has 100 Obstetric beds and has five units each of which runs an antenatal clinic one day a week with an average clinic attendance of 150 per clinic [23]. It also has an emergency unit which provides a 24-h service on a daily basis. The total monthly deliveries are about 250–300. The study was conducted from June 2017 to July 2018. This study was registered with the Pan African Clinical Trials Registry (PACTR) on April 19, 2023 (Reference Number: PACTR202304579281358).

Pregnant women were included if they were at 34–36^+6^ weeks’ gestation and were at risk of imminent premature delivery (either spontaneously or if early delivery was recommended because of fetal or maternal indications) at the time of admission to the hospital. Gestational age was defined according to the first day of the woman’s last menstrual period, if known and reliable, or by early ultrasonography carried out below 20 weeks of gestation if the last menstrual period was not known. All consenting pregnant women of gestational age 34 + 0 and 36 + 6 weeks, who were at high risk of delivering before 37 weeks were included in the study. A high probability of preterm delivery includes women with intact membranes and at least 3 cm dilation or 75% effacement of the cervix, spontaneous rupture of the membranes, expected preterm delivery for any other indication by induction of labor or caesarean section [6].

### Sample size

To determine the minimum sample size estimate for this study, the Corlien method of sample size estimation for interventional studies was used:

$${\text{n}}=\frac{{(\mathrm{\alpha }+\upbeta )}^{2} [{{\text{P}}}_{1} (1-{{\text{P}}}_{1}) +{{\text{P}}}_{2} (1-{{\text{P}}}_{2})}{{({{\text{P}}}_{2}-{{\text{P}}}_{2})}^{2}}$$

n = Minimum sample size.

P_1_ = Prevalence of preterm infants with respiratory distress = 28.9% [24].

P_2_ = Expected prevalence of preterm infants with respiratory distress after corticosteroid administration = 14.5%

α = percentage point of the normal distribution corresponding to the two sided significant level of 5% = 1.96.

β = power to detect a 50% reduction in the prevalence of respiratory disorders following an administration of corticosteroid to the study group is 80% = 0.84

$${\text{n}}= \frac{{(1.96+0.84)}^{2} [0.289(0.771) +0.145(0.855)}{{(0.144)}^{2}}$$

$${\text{n}}= \frac{(7.84) (0.223 + 0.124)}{0.021}=\frac{2.72048}{0.021}$$

$${\text{n}}=129.54$$

Hence, the calculated minimum sample size obtained for each group was 130.

Assuming a drop out rate of 10% = 13.

13 was as added to the minimum sample size of 130 = 143

N = 143 in each group.

Therefore, the minimum sample size was 286.

## Inclusion criteria

All consenting pregnant women at the General Hospital Ifako Ijaye with gestational age between 34 + 0 and 36 + 6 weeks, who were at high risk of delivering before 37 weeks.

A high probability of preterm delivery includes:

Women with intact membranes and at least 3 cm dilation or 75% effacement of the cervix.

Spontaneous rupture of the membranes.

Expected preterm delivery for any other indication by induction of labour or caesarean section.

## Exclusion criteria

All pregnant women with multiple pregnancy.

Suspected or diagnosed major congenital malformations.

Uncertain gestational age.

Clinical evidence of chorioamnionitis.

Previous corticosteroid use during the antenatal period.

A woman who is expected to deliver in less than 12 h.

### Randomization and follow-up

There was a random allocation sequence of all the serial numbers assigned to all the eligible and consenting women in a 1:1 ratio by an independent statistician. The table of random numbers was prepared in a single block using random allocation software (version 2.0) in which 143 women were randomized to receive dexamethasone and 143 were given placebo. Two hundred and eighty-six drug envelopes containing either 2 doses of dexamethasone or placebo which were identical in appearance, volume and colour were prepared by a member of the pharmacy department and were numbered using the random allocation sequence generated. Thus, the drugs (i.e. dexamethasone and placebo) were packaged and numbered based on the random allocation sequence generated by the independent statistician. Since the patient presented to the hospital in batches, they were numbered serially as they presented at the hospital.

The hospital pharmacist who was responsible for packing the drugs and placebo withdrew 12 mg of dexamethasone (3mls) into 5 ml syringes and withdrew 3mls of 0.9% saline solution into 5 ml syringes. Each drug envelope contained 2 of such 5 ml syringes containing either dexamethasone or 0.9% saline solution, and each participant was given the content of one 5 ml syringe initially and the other 5 ml syringe in the drug envelope 12 h later.

Only the hospital pharmacist responsible for packing the drugs into the drug envelopes was aware of their contents. The investigator, the obstetrician who cared for the women, and the women were not aware of the content of the drug envelopes.

Women who met the inclusion criteria were identified and were given an informed consent form to sign. They received the sealed envelope corresponding to their randomization number and the drug contained in the envelope was administered by a trained research assistant at the level of a house officer cadre. Three ampoules were applied intramuscularly, with three more administered 12 h later. After administering the medications, all the pregnant women were subsequently managed based on the standard treatment protocol of the labour ward of the hospital. In the case of patients scheduled for induction of labour or elective caesarean section, this was planned such that the procedure commenced not before 24 h after the administration of the drug.

The investigators followed up the women prospectively and collected data on the pregnant women and their newborns on a standardized form. An independent neonatologist who was also blinded from the treatment arms assessed the neonates for acute respiratory distress syndrome and/or Transient Tachypnea of the Newborn based on the clinical signs, symptoms, chest x-ray results and other blood investigation results (where indicated to rule out other similar diagnosis) and the trained research assistant entered his/her findings in the proforma.

Women who were discharged from the hospital while still pregnant and who went on to deliver elsewhere were excluded from the study after randomization. When the study was completed, the research team were then unblinded to the serial numbers in each block (cases and controls) in the random allocation sequence.

### Ethical considerations

Permission to conduct the study was sought from the Head, Obstetrics and Gynaecology department, Lagos State University Teaching Hospital, Ikeja. Ethical approval to conduct the study was sought from the ethical committee of the Lagos State University Teaching Hospital (LASUTH) Health Research and Ethical Review Committee. Informed consent was obtained from the pregnant women who agreed to participate in the study. Those who chose not to participate did not have their treatment affected in any way. Confidentiality and anonymity were maintained throughout the study using serial numbers only.

### Data management

The data were entered into the computer and analyzed using Epi Info 3.5.3 statistical software for the centre for disease control and prevention Atlanta USA. The results were presented using appropriate frequency tables and charts while measures of central tendencies (mean or median) and measures of dispersion (standard deviation and range) were calculated for quantitative variables.

Statistical associations between categorical variables were tested using Pearson's chi-square test or Fisher test as appropriate. Continuous variables were compared using the student T test or Anova as appropriate and logistic regression analysis was used to identify the predictors of the outcome of the neonatal health status. Level of statistical significance was set at *p*-value less than 5%.

## Measurement of key variables

### Primary outcome variables

Presence of neonatal respiratory morbidity. These include respiratory distress syndrome; Transient tachypnea of the newborn and the need for ventilatory support within 72 h of delivery with supplemental oxygen for at least 2 h. Any neonate who had any of the 3 conditions is said to have had respiratory morbidity.

### Secondary outcome variables

These include type of delivery; gestational age at birth; Apgar Scores at first and fifth minutes; admission to the Special Care Baby Unit (SCBU); neonatal hypoglycemia; neonatal jaundice; neonatal sepsis; other neonatal morbidities; length of stay at the hospital; neonatal death within 72 h of delivery; the need for resuscitation at birth and feeding difficulties.

## Definitions

### Respiratory distress syndrome

Presence of clinical signs of respiratory distress (tachypnea, chest wall retraction, flaring of the alae nasi, grunting or cyanosis), presenting within hours after birth, most often immediately after delivery, with a requirement for supplemental oxygen and a chest x ray showing reduced air entry and reticulogranular infiltrates.

### Transient tachypnea of the newborn

Tachypnea in the absence of chest x ray or with a chest x ray showing normal or increased perihilar interstitital markings resolving within 72 h.

## Results

A total of 286 women fulfilled the inclusion criteria for the study. 143 were randomized to receive dexamethasone (cases) and 143 were randomized to receive placebo (controls). Of these, 34 women were discharged from the hospital during pregnancy and were lost to follow-up, 13 among the cases and 21 among the controls, therefore 130 women remained in the dexamethasone group and 122 in the placebo group with 11.9% of losses after randomization.

Table 1 shows the socio demographic characteristics of the participants. The mean maternal age of the cases is 31.2 ± 4.8 years while that of the controls is 30.1 ± 4.9 years. These two mean maternal ages showed no statistically significant difference (*p* = 0.55). With respect to occupation, there was no significant difference between the cases and the controls (*p* = 0.35). There were no significant differences noted also in the religious beliefs, tribe, and highest level of education of the participants.

**Table 1:** Sociodemographic characteristics of the cases and controls

Variables	Cases *N* = 130	Control *N* = 122	χ^2^ (*p*-value)
**Maternal age (mean ± SD)**	31.2 ± 4.8	30.1 ± 4.9	0.55
**Occupation**			
Skilled	83(52.9)	74(47.1)	1.181(0.55)
Semi-skilled	30(46.2)	35(53.8)	
Unskilled	17(56.7)	13(43.3)	
**Highest level of education**			
Primary and below	5(41.7)	7(58.3)	2.105(0.35)
Secondary	33(45.8)	39(54.2)	
Tertiary	92(54.8)	76(45.2)	
**Tribe**			
Hausa	14(45.2)	17(54.8)	(2.729)0.44
Igbo	47(53.4)	41(46.6)	
Yoruba	55(55.6)	44(44.4)	
Others	14(41.2)	20(58.8)	
**Religious belief**			
Christianity	76(49.4)	78(50.6)	(1.609)0.44
Islam	53(54.6)	44(45.4)	
Traditional	1(100.0)	0(0.0)	

Table 2 shows the obstetric characteristics of the participants, 59.5% of the cases were booked while 40.5% of the controls were booked and this difference was statistically significant (*p* = 0.02). There was no statistically significant difference in the parity, birth weight of the fetus and gestational age at delivery of the cases compared to the controls.

**Table 2:** Obstetric characteristics of the cases and controls

Variable	Cases *N* = 130	Control *N* = 122	*P* value
**Booking status**			
Booked	69(59.5)	47(40.5)	**0.02**
Unbooked	61(44.9)	75(55.1)	
Parity	1(0,2)^a^	1(0,2)^a^	0.55
Birth weight in grammes	2974.61(± 453.29)	2945.90(± 561.15)	0.65
GA at Delivery	35.68(± 0.93)	35.75(± 0.94)	0.51

^a^Median(inter quartile range)

Table 3 shows the Incidence of respiratory morbidity among the cases and controls. Any neonate who had any of the 3 primary outcome measures vis respiratory distress syndrome, transient tachypnea of the newborn or need for ventilatory support is said to have had a respiratory morbidity. The risk for the occurrence of at least one type of respiratory morbidity was significantly lower in those that received corticosteroid compared to those that did not receive. (OR = 0.12, 95% C.I = 0.04 – 0.31, *P* = 0.00003). Corticosteroid administration was associated with significant reduction in the rate of respiratory distress syndrome (O.R = O.27, 95% C.I = 0.08 – O.84, *p* = 0.032), Transient tachypnea of the newborn (O.R = 0.11, 95% C.I = 0.02 – 0.49, *p* = 0.0016), and need for ventilatory support (O.R = 0.15, 95% C.I = 0.05 – 0.39, *p* = 0.00006).

**Table 3:** Incidence of respiratory morbidity among cases and controls

Variables	Cases *N* = 130	Control *N* = 122	Odds ratio	95% C.I	*p*-value
**Respiratory Morbidity**					
Yes	5(3.8)	31(25.4)	0.12	0.04 – 0.31	0.000003^*^
No	125(96.2)	91(74.6)	Ref	Ref	
**Respiratory distress syndrome**					
Yes	3(3.1)	13(10.7)	0.20	0.06—0.69	0.032^*^
No	127(96.9)	109(89.3)	Ref	Ref	
**Transient tachypnea of newborn**					
Yes	2(1.5)	15(12.3)	0.11	0.02 – 0.49	0.0016^*^
No	128(98.5)	107(87.7)	Ref	Ref	
**Need for ventilatory suppor**					
Yes	5(3.8)	26(21.3)	0.15	0.05—0.39	0.00006^*^
No	125(96.2)	96(78.7)	Ref	Ref	

^*^Significant *p*-value

Table 4 shows the association of other non-respiratory neonatal complications with dexamethasone use. Corticosteroid administration did not significantly affect the risk of hypoglycaemia, neonatal sepsis, neonatal jaundice, neonatal death and feeding difficulties. Corticosteroid use however significantly reduced the risk of birth asphyxia (O.R = 0.25, 95% C.I = 1.57 – 10.47, *p* = 0.004), admission to neonatal intensive care unit (O.R = 0.41, 95% C.I = 0.21 – 0.78, *p* = 0.009) and need for active resuscitation at birth (O.R = 0.31, C.I = 0.12 – 0.76, *p* = 0.014).

**Table 4:** Bivariate analysis of other neonatal complications among cases and controls

Variables	Cases *N* = 130	Controls *N* = 122	Odds ratio	95% C.I	*p*-value
**Birth Asphyxia**					
Yes	6(4.6)	20(16.4)	0.25	0.10 – 0.63	0.004*
No	124(95.4)	102(83.6)	Ref	Ref	
**NICU admission**					
Yes	17(13.1)	33(27.0)	0.41	0.21—0.78	0.009*
No	113(86.9)	89(73.0)	Ref	Ref	
**Hypoglycemia**					
Yes	10(7.7)	10(8.2)	0.93	0.38 – 2.28	0.93
No	120(92.3)	112(91.8)	Ref	Ref	
**Neonatal sepsis**					
Yes	10(7.7)	11(9.0)	0.84	0.35 – 2.02	0.88
No	120(92.3)	111(91.0)	Ref	Ref	
**Neonatal jaundice**					
**Yes**	9(6.9)	10(8.2)	0.83	0.33 – 2.08	0.89
**No**	121(93.1)	112(91.8)	Ref	Ref	
Neonatal death					
Yes	2(1.5)	4(3.3)	0.46	0.10—2.21	0.62
No	128(98.5)	118(96.7)	Ref	Ref	
Need for resuscitation at birth					
Yes	7(5.4)	19(15.6)	0.31	0.13 – 0.75	0.014
No	123(94.6)	103(8.4)	Ref	Ref	
Feeding difficulties					
Yes	1(0.8)	3(2.5)	0.31	0.04– 2.22	0.58
No	129(99.2)	119(97.5)			

Table 5 shows the association between maternal factors and the occurrence of respiratory morbidity in the neonate. The presence of hypertension, preterm labour, pre labour rupture of membranes and antepartum haemorrhage had no significant effect on respiratory morbidity.

**Table 5:** Bivariate association between maternal factors and effect on respiratory morbidity

Variable	Respiratory morbidity		Odds Ratio	95% C.I	*P* value
	Yes (*n* = 36)	No (216)			
	**Yes**	**No**			
**Route of delivery**					
Caesarean section	24(18.6)	105(81.4)	2.11	1.01 – 4.43	0.04
Vagina delivery	12(9.8)	111(90.2)	Ref		
**Hypertension**					
Yes	13(15.3)	72(84.7)	1.13	0.54 – 2.36	0.89
No	23(13.8)	144(86.2)	Ref		
**Diabetes Mellitus**					
Yes	8(36.4)	14(63.6)	4.12	1.59 – 10.71	0.0054
No	28(12.2)	202(87.8)	Ref		
**Preterm Labour**					
Yes	2(5.3)	36(94.7)	0.29	0.07 – 1.26	0.14
No	34(15.9)	180(84.1)	Ref		
**Pre-labour rupture of membranes**					
Yes	2(4.9)	39(95.1)	0.27	0.06 – 1.14	0.10
No	34(16.1)	177(85.1)	Ref		
**Ante partum haemorrhage**					
Yes	6(11.8)	45(88.2)	0.76	0.30 – 1.92	0.72
No	30(14.9)	171(85.1)	Ref		

Delivery by caesarean section compared to vaginal delivery (O.R = 2.11, 95% C.I of 1.01 – 4.44, *p* = 0.04) and presence of diabetes mellitus (O.R = 4.12, 95% C.I = 1.59 – 10.71, *p* = 0.0054) significantly increased the risk of respiratory morbidity.

Table 6 shows the multiple logistic regression analysis of factors which had significant effects on respiratory morbidity. After correcting for presence of diabetes mellitus and delivery by caesarean section, corticosteroid administration to the mother at risk of late preterm delivery reduced the risk of respiratory morbidity almost ninefold (AOR = 0.11, 95% C.I = 0.04 – 0.31, *p* = < 0.00001).

**Table 6:** Multiple logistic regression analysis of corticosteroid administration as an independent determinant of respiratory morbidity

Variable	AOR	95% C.I	*p* value
Corticosteroid administered	0.11	0.04 – 0.31	0.0000
Diabetes mellitus	4.46	1.50—13.29	0.0071
Ceasarean section	2.12	0.96- 2.16	0.0626

*AOR* Adjusted odds ratio

## Discussion

This placebo controlled, randomized controlled trial, evaluated the efficacy of antenatal corticosteroids (dexamethasone) in reducing respiratory morbidity in neonates delivered in the late preterm period. This trial shows that treatment with antenatal corticosteroids in women at 34–36^+6^ weeks of gestation at risk of having preterm delivery is effective in reducing the occurrence of respiratory disorders in their babies, reducing the risk almost ninefold of those without steroids. Dexamethasone administration resulted in reduced rates of respiratory distress syndrome, transient tachypnea of the newborn, need for ventilatory support within the first 72 h of life, need for active resuscitation at birth and hence, need for NICU admission.

This study revealed that there was a significant threefold reduction in the incidence of respiratory distress syndrome among the participants. This finding is consistent with the findings of a previous study by Serrano et al. in Spain [14]. In this prospective observational study, it was reported that betamethasone administration was significantly associated with a reduction in the level of respiratory distress syndrome among the parturient [14].

In this study, there was a ninefold reduction in the level of transient tachypnea of the newborn among the neonates whose mothers had antenatal corticosteroid administered as against the controls. This finding is similar to that reported by Gyamfi-Bannerman et al. in the United States of America who reported approximately a twofold increase in the level of transient tachypnea of the newborn among the neonates of the parturients who were not given antenatal corticosteroid [16]. However, Porto et al. in Brazil did not find any significant association between corticosteroid administration and reduction in the level of transient tachypnea of the newborn among the neonates [17]. A study conducted in Thailand in 2015 also failed to demonstrate a significant reduction in the level of transient tachypnea of the newborn with corticosteroid administration [12]. The difference between the findings of this study and those of the previous studies [12, 17] might be due to inherent racial differences associated with fetal lung maturity.

Consistent with the finding of this study is that of the Antenatal Betamethasone for Women at risk of Late Preterm delivery (ALPS) trial, which studied 2831 women who had a high probability of delivery in the late preterm period. These women were randomly assigned to receive either antenatal betamethasone or placebo. The study reported a significant reduction in the rate of admission to neonatal intensive care units for respiratory complications and the need for active resuscitation at birth in the betamethasone group, as compared to the controls [25].

Also, the antenatal late preterm steroid trial, a double blind, placebo controlled, randomized controlled trial found a very significant reduction in the need for respiratory support within the first 72 h among the participants who had antenatal corticosteroid administered as compared to the controls [16]. There was also a significant reduction in the rates of severe respiratory morbidity, bronchopulmonary dysplasia, transient tachypnea of the newborn, the need for resuscitation at birth and the need for postnatal surfactant [16].

The administration of dexamethasone also had a significant difference in the rate of NICU admission among the dexamethasone and placebo group. Studies have shown that respiratory distress is one of the commonest indications for NICU admission [26, 27]. In an Iranian study done in 2011, the most common cause of intensive care admission was respiratory distress and was the most common cause of death alone or concomitant with malformation or sepsis [27]. It is therefore not surprising that administration of antenatal corticosteroid was associated with a reduction in the rate of NICU admission since it caused a significant reduction in the occurrence of respiratory morbidity. This finding is worthy of note especially in developing countries like Nigeria where neonatal care is sub-optimal and not readily available especially in the rural parts of the country, and where it is available, it is very expensive and not readily affordable [28]. Antenatal corticosteroids by reducing neonatal respiratory morbidity and NICU admissions, will probably help in saving cost and the stress on our health facilities.

This study found no statistically significant difference in the rates of hypoglycemia, gestational age at delivery, neonatal sepsis, neonatal jaundice, neonatal deaths and feeding difficulty. These findings are consistent with the findings from other studies [16, 17].

The study also looked at the effect of maternal factors like diabetes mellitus, hypertension, pre-labour rupture of membranes, antepartum haemorrhage, and mode of delivery i.e. caesarean section on the occurrence of respiratory morbidity in the neonates. Diabetes mellitus and caesarean section increased the risk of respiratory morbidity, and this finding is not surprising as several studies have demonstrated similar findings [29–31]. Caesarean delivery has been shown to be a risk factor for neonatal respiratory morbidity with lack of the physiological catecholamine surge and fluid retention in the lungs being the most likely causes. Recent evidence indicates that molecular mechanisms (lung epithelial sodium channels) promote alveolar fluid drainage, and these channels may be underactive in fetuses not exposed to the process of labor [32]. Also, the process of vaginal squeeze during vaginal delivery helps to remove the excess fluid from the fetal lungs [30].

These two factors (diabetes mellitus and caesarean section) were put into the logistic regression model, corticosteroid still retained its significance and reduced the risk by 90% Hence, corticosteroid on its own has an independent effect in reducing respiratory morbidity.

This study is limited by the fact that it is an institution-based study with a relatively small sample size which may not be representative of the general population, thus a larger scale multi centered study will be required to evaluate the benefit in the general populace. Despite this however, it has shown that the administration of antenatal corticosteroid to pregnant women at risk of having late preterm delivery reduces the risk of neonatal respiratory morbidity by 9 folds and the need for NICU admission. This could help save cost and the stress on our health facilities. It also reduces other neonatal complications such as birth asphyxia and need for active resuscitation at birth. These findings are not likely to be different in other parts of the country. Also, risk factors of the disease such as the history of a previous affected sibling and the sex of the baby were not included in the exclusion criteria. Furthermore, as current research suggests a more restrictive use of antenatal corticosteroids as they have been implicated in an increase in neurodevelopmental disorders in childhood, further studies are needed in the Nigerian population to examine the long term effect on these babies.

## Data Availability

The datasets generated from this current study are available from the corresponding author upon reasonable request.
